# Supplementary figures and images for: NF-kappa-B activation unveils the presence of inflammatory hotspots in human gut xenografts
Source: PLoS One. 2021 May 3;16(5):e0243010. doi: 10.1371/journal.pone.0243010 (PMC8092666; doi:10.1371/journal.pone.0243010)

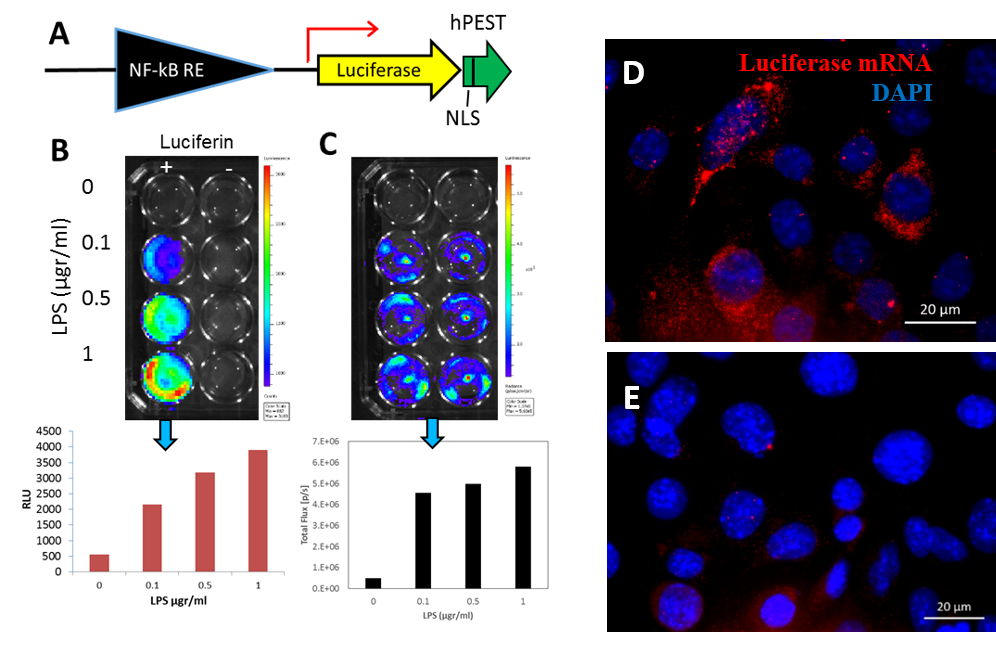

Supplement: S1 Fig — (A) demonstrating expression and transcription of luciferase using luminescence analysis (B-C) and fluorescence in situ hybridization (FISH; D-E) of luciferase (luc2CP) mRNA. Human THP-1 (B) and mouse RAW 264.7 (C) macrophage-like cell lines, were infected with the lentiviral NF-κB reporter vector (A). Cells were activated with LPS (B-C), and Mycoplasma bovis lipoproteins (D). Luminescence imaging and analysis were performed using cooled CCD optical macroscopic imaging system (B-C; IVIS Lumina Series III, PerkinElmer Inc., MA USA). FISH and nuclear staining with 4,6-diamidino-2-phenylindole (DAPI) of activated (C) and control EPH4 cells (D) demonstrating luc2CP transcripts in activated cells. Microscopic images were taken with epifluorescence microscopy system (Axio Imager M1, Zeiss, Germany). Scale bars 20 μm (C-D). (TIF) [file pone.0243010.s001.tif]

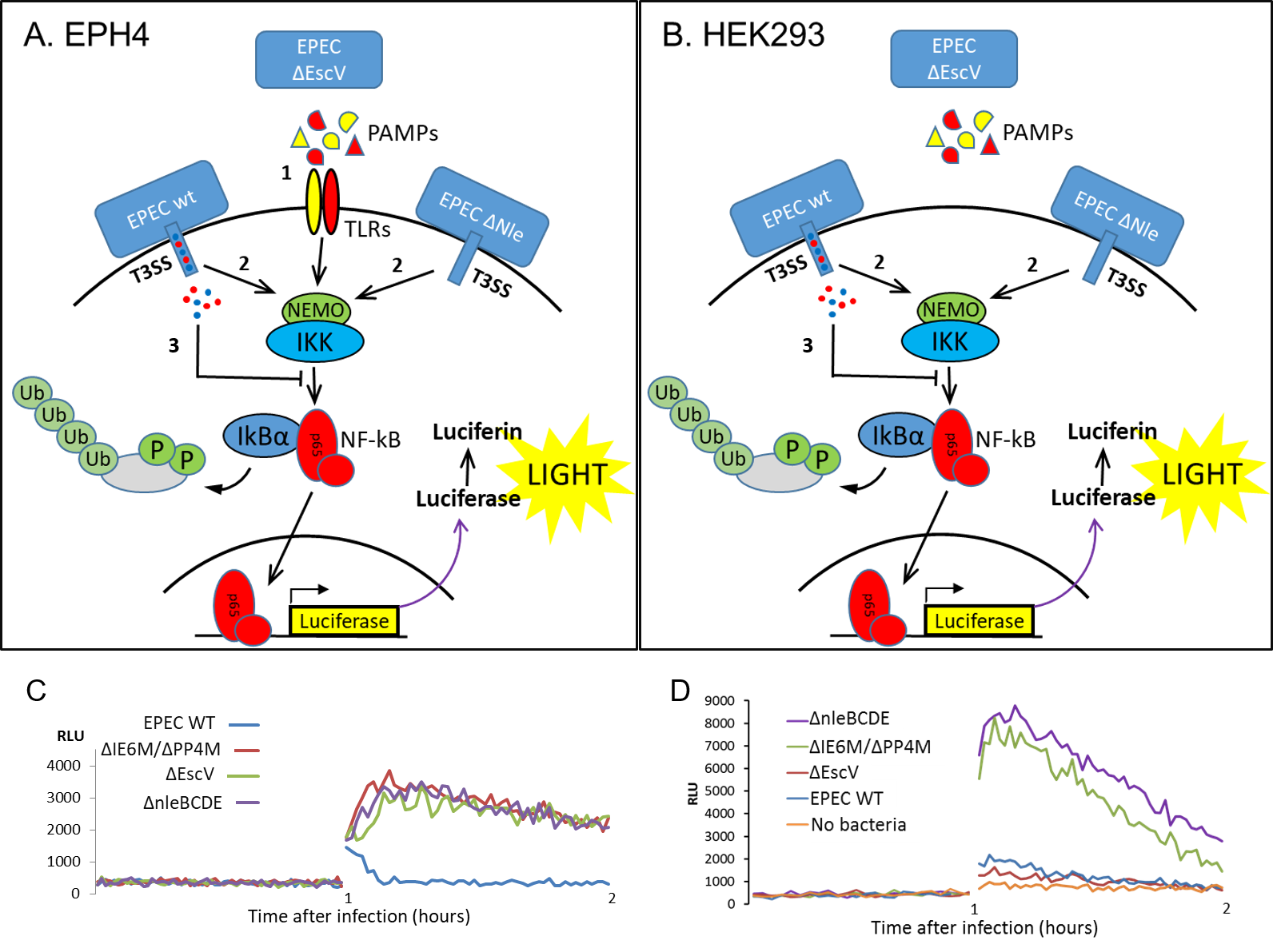

Supplement: S2 Fig — Mouse mammary epithelial line EPH4 (A&C) and human embryonic cell line HEK293T (B&D) were infected with the lentiviral NF-κB reporter vector (S1A Fig). Transduced cells were infected with wild type (WT) enteropathogenic E. coli bacteria, the type 3 secretion system (T3SS)-defective mutant strain ΔescV, and ΔIE6/PP4, and ΔnleBCDE mutant strains which are defective in anti-NF-κB effectors. In EPH4 cells NF-κB pathway is activated by all bacterial strains through pathogen associated molecular patterns (PAMPs)-toll-like receptor (TLR) signalling (1 in A) and T3SS-dependent activation following infection with WT and the anti- NF-κB-defective mutant strains (2 in A). Inhibition of NF-κB activation is mediated by T3SS-dependent anti- NF-κB effectors and is limited to WT infection of EPH4 cells (3 in A). HEK293T cells are TLR signaling-defective and both activation (2 in B) and inhibition (3 in B) of NF-κB signaling is T3SS-dependent. Luminescence analysis of NF-κB activation following infection with the above described bacterial strains were performed using SpectraMax i3x multiple detection microplate reader (Molecular Devices, CA USA) and are presented in C-D. Representative results of one experiment out of three similar experiments. (TIF) [file pone.0243010.s002.tif]

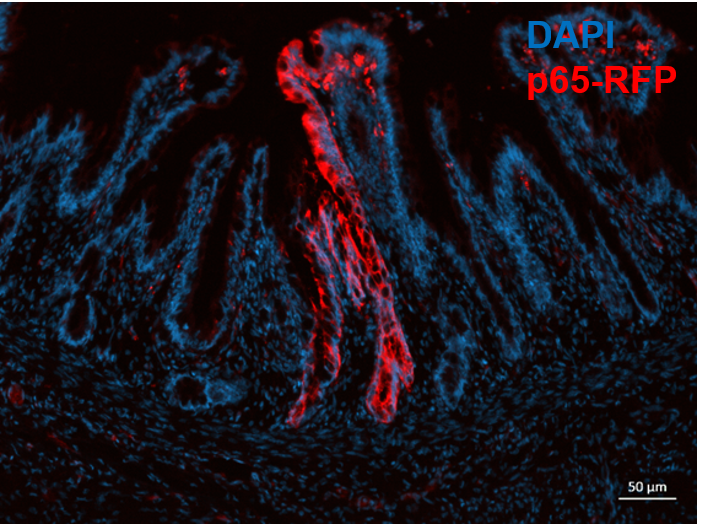

Supplement: S3 Fig — Human fetal gut was infected with lentivirus carrying the human p65-RFP gene. Fetal gut was transplanted subcutaneously in SCID mouse and allowed to develop over 12 weeks into a mature pediatric gut. Cryosections of the gut xenograft were stained with DAPI imaged under fluorescence microscopy. The heritable genetic marker was integrated into stem cells via lentiviral vector infection, all other cells transduced by the lentivirus vector were lost in the course of xenograft development over a 12 week period. The transduced stem cells in which the p65-RFP transgene have been integrated generated progeny that produce the p65-RFP protein. These cells extend from the crypt base to the villus tip and can be visualized as ribbons of red fluorescing cells. (TIF) [file pone.0243010.s003.tif]
